# Physicochemical, Bacteriostatic, and Biological Properties of Starch/Chitosan Polymer Composites Modified by Graphene Oxide, Designed as New Bionanomaterials

**DOI:** 10.3390/polym13142327

**Published:** 2021-07-15

**Authors:** Magdalena Krystyjan, Gohar Khachatryan, Maja Grabacka, Marcel Krzan, Mariusz Witczak, Jacek Grzyb, Liliana Woszczak

**Affiliations:** 1 Faculty of Food Technology, University of Agriculture in Krakow, Balicka Street 122, 30-149 Krakow, Poland; gohar.khachatryan@urk.edu.pl (G.K.); maja.grabacka@urk.edu.pl (M.G.); mariusz.witczak@urk.edu.pl (M.W.); liliana.woszczak@urk.edu.pl (L.W.); 2 Jerzy Haber Institute of Catalysis and Surface Chemistry, Polish Academy of Sciences, 31-120 Krakow, Poland; marcel.krzan@ikifp.edu.pl; 3 Faculty of Agriculture and Economics, University of Agriculture in Krakow, Mickiewicza 24/28, 30-059 Krakow, Poland; jacek.grzyb@urk.edu.pl

**Keywords:** bionanomaterials, polymers, graphene oxide, chitosan, starch

## Abstract

The application of natural polymer matrices as medical device components or food packaging materials has gained a considerable popularity in recent years, this has occurred in response to the increasing plastic pollution hazard. Currently, constant progress is being made in designing two-component or three-component systems that combine natural materials which help to achieve a quality comparable to the purely synthetic counterparts. This study describes a green synthesis preparation of new bionanocomposites consisting of starch/chitosan/graphene oxide (GO), that possess improved biological activities; namely, good tolerability by human cells with concomitant antimicrobial activity. The structural and morphological properties of bionanocomposites were analyzed using the following techniques: dynamic light scattering, scanning and transmission electron microscopy, wettability and free surface energy determination, and Fourier transform infrared spectroscopy. The study confirmed the homogenous distribution of GO layers within the starch/chitosan matrix and their large particle size. The interactions among the components were stronger in thin films. Additionally, differential scanning calorimetry analysis, UV–vis spectroscopy, surface colour measurements, transparency, water content, solubility, and swelling degree of composites were also performed. The mechanical parameters, such as tensile strength and elongation at break (EAB) were measured in order to characterise the functional properties of obtained nanocomposites. The GO additive altered the thermal features of the composites and decreased their brightness. The EAB of composite was improved by the introduction of GO. Importantly, cell-based analyses revealed no toxic effect of the composites on HaCat keratinocytes and HepG2 hepatoma cells, although a pronounced bacteriostatic effect against various strains of pathogenic bacteria was observed. In conclusion, the starch/chitosan/GO nanocomposites reveal numerous useful physicochemical and biological features, which make them a promising alternative for purely synthetic materials.

## 1. Introduction

Nanotechnology has currently been attracting attention due to its appreciable role in the agricultural and food industries, where it is innovatively used to improve food quality and safety. The unique physical, chemical, and biological features of nanomaterials facilitate their application in medical and biological sciences, cosmetology, waste water treatment, and other fields [1,2]. In recent years, nanotechnology has also been introduced in packaging systems, where the environmental friendliness of packaging material, product shelf-life, sensory quality, and safety are the most important factors for the food industry. Modern active food packaging contributes to the control of quality, temperature stability and microbiological safety [2]. The increasing number of antibiotic-resistant microbes, as well as oxidative processes, present major challenge for the food packaging sector. Traditional packaging materials do not always meet the requirements for adequate food protection and preservation, or they are not environmentally friendly. Whereas active packaging provides the possibility of microbiological control, and so is an important tool in reducing food wastage and loss [3]. Active packaging also helps to prevent the spread of food-borne diseases. This is the rationale for the application of nanotechnology in food production, processing, storage, and distribution [2]. 

Polysaccharides, due to the diversity of functional groups, are a particularly rewarding material for modification; furthermore, their low cost and abundance in natural resources makes them attractive for industrial applications. Moreover—being biodegradable, renewable, and most importantly nontoxic—polysaccharides are likely to successfully compete with synthetic polymers in the near future. Starch and chitosan belong to the biopolymers most frequently used in the production of sustainable food packaging. Chitosan is regarded as a biocompatible material, and its proven antimicrobial activity makes it suitable for medical, therapeutic, and pharmaceutical applications in drug delivery, tissue engineering, orthopaedic and periodontal applications, as well as wound healing [3,4]. Chitosan is also used as the scaffold material in hydrogels [5] as well as the packaging material for the quality preservation of a variety of food products [6]. It has been confirmed that, in comparison to other bio-based materials, chitosan has the advantage of being able to incorporate functional substances such as vitamins or minerals [6,7]. Despite numerous advantages, polysaccharide composites have some limitations, which means that when used individually, they cannot compete with synthetic materials, mainly due to their low barrier properties and high susceptibility to mechanical damage. Many attempts have been made to improve their properties—e.g., by adding plasticisers or hydrophobic materials [8,9,10]. It seems, however, that the production of composites consisting of several polymers, including graphene, may help to achieve promising results. Graphene oxide has an amphipathic character due to the combination of a hydrophobic honeycombed 2D carbon structure with hydrophilic hydroxyl and carboxylic groups on its edges. The presence of surface polar groups facilitates the GO interactions with biomolecules (e.g., proteins and lipids) and determines its unique properties: large planar surface, electron delocalisation, lightweight, high Young’s modulus, high thermal and electrical conductivity, good diffraction strength, mobility of charge carriers and biocompatibility [11,12,13,14,15]. Such characteristics encourage a broad range of applications in bioimaging, drug and gene delivery, and formation of tissue scaffolding, as well as in antimicrobial materials [16]. The antimicrobial feature of graphene and graphene-derived materials encourages medical use as an alternative to classic antibiotics [11]. Despite all these advantages, the low solubility of graphene nanoparticles and a tendency to aggregate due to strong inter-planar attractive forces, hampers its antibacterial applications. The smaller size of nanoparticles improves graphene antibacterial activity. In such a form, GO could be introduced into a polymer matrix in order to enhance the antimicrobial potential of active packaging technology. The goal is to achieve a stable nanocarbon–polymer dispersion that would be resistant to aggregation [17,18,19]. This was the main objective of our study. 

A survey of the most recent literature indicates that graphene-containing bionanocomposites are very promising materials in the construction of biosensors, drug delivery systems, modified electrodes, energy-storage systems, and active packaging [20]. Jamróz et al. [21] demonstrated that nanocomposite films based on furcellaran (FUR) and nanofillers (GO) might be used as UV-blocking materials. The authors confirmed that the addition of GO improved tensile strength, but concomittantly reduced water content, solubility and elongation at break of the FUR-composites. Lee and Mahajan [22] described the possibility of using graphene as a biosensor. A very interesting invention was reported by Yang et al. [23] who applied highly absorptive β-cyclodextrin modified GO composites to remove organic dyes from wastewater. Ma et al. [24] synthesised graphene oxide–cerium oxide (GO–CeO2) hybrids through an in situ hydrothermal approach and incorporated them into epoxy resin to prepare a robust coating for aluminium alloy protection. Lyn et al. [25] prepared active packaging from chitosan (CS) incorporated with GO to maintain the quality and extend the storage life of palm-oil based margarine. Their innovative composites protected products from UV and had remarkable antioxidant features important for food protection. Starch has also been used in an innovative packaging design. Narayanan et al. [26] used soluble starch biopolymer as a functionalising and reducing agent for the preparation of starch-reduced graphene oxide (SRGO). 

Drawing inspiration from all these innovative solutions we aimed to exploit the unique properties of potato starch and chitosan. We combined these two polymers into a matrix for the introduction of graphene oxide to obtain a bionanocomposites material with improved functional (i.e., antimicrobial) and mechanical properties, which would hopefully broaden the range of possible applications, for instance in food packaging and preservation. According to European Union regulations, packaging and all other materials intended to have contact with food, need to be sufficiently inert not to release any exogenous substances in a quantity that could adversely affect human health, food composition, or its organoleptic characteristics [27]. Therefore, we aimed to assess any potentially negative effects of GO on the viability of human cell cultures of skin keratinocyte HaCat and hepatocyte-derived HepG2 cell lines.

## 2. Materials and Methods

### 2.1. Materials 

Potato starch (PS) was purchased from PPZ Bronisław company (Strzelno, Poland). This particular starch contained 19.90% moisture and 26.48% amylose. Chitosan (Ch) from shrimp shells was purchased from Sigma (CAS no 9012-76-4). Graphene oxide (GO) was purchased from NANOMATERIALS Leszek Stobiński (Warsaw, Poland). Glycerol—used as a plasticiser—was purchased from F.H.U. DOR-CHEM (Cracow, Poland).

### 2.2. Preparation of Composites and Nanocomposites

The composites were made of starch and chitosan with the addition of graphene oxide. First, a 2% chitosan gel (Ch) was prepared by adding 30 g of chitosan to 1470 g of acetic acid solution (0.5%), stirred at 70 °C until a clear gel was obtained. Then a 4% starch gel (PS) was prepared by adding 30 g of potato starch into 720 g of distilled water with constant mixing at 80 °C for 30 min.

Control Composite (Composite C)

The control composite was prepared from starch and chitosan gels: 500 g of Ch gel was added to 250 g of PS gel and stirred approx. 30 min, until a homogeneous gel was obtained. Then 10 g of glycerol (half the weight of the polysaccharides) and 25 g of distilled water were added. The mixture was stirred for 30 min and poured into plates to dry.

Nanocomposite I

Nanocomposite I consisted of PS/Ch with GO: 500 g of Ch gel and 250 g of PS gel were mixed together for 30 min. Then, 10 g of glycerol (a plasticiser) and 25 g of GO (0.1%, *v/v*) were added. Next, the mixture was stirred for 30 min and poured into plates to dry.

Nanocomposite II

**Figure 1:**
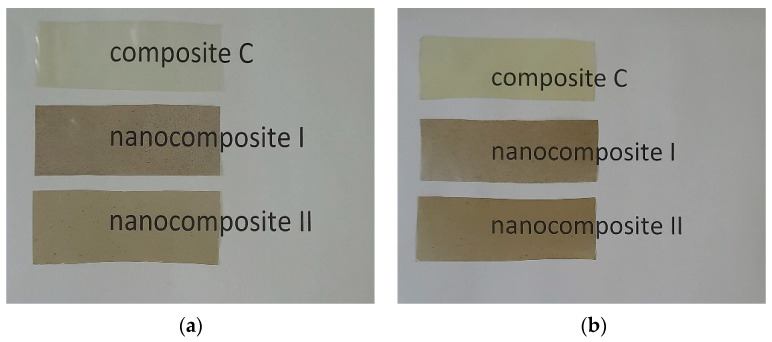
The obtained composites put on their printed names: (**a**) thin and (**b**) thick composites.

Nanocomposite II consisted of PS/Ch gels with half of the GO amount: 500 g of Ch gel and 250 g of PS gel were mixed together for 30 min. Then, 10 g of glycerol and 12.5 g of GO (0.1%, *v/v*) and 12.5 g of H_2_O were added. The mixture was stirred for 30 min and poured into plates to dry. All samples (Figure 1) (differing in the graphene content and the film thickness) were dried in an oven at 40 °C for 2 days to obtain films.

### 2.3. Dynamic Light Scattering (DLS) Measurements of Zeta Potential and Particle/Aggregate Sizes

The zeta potential and particle/aggregate sizes were measured using Malvern Zetasizer Nano ZS apparatus with disposable measurement cells (DTS 1065, Malvern). Zeta potential was calculated from the electrophoretic mobility of particles using the Smoluchowski model. The results are expressed as an average from three consecutive measurements with 20 runs.

### 2.4. Wettability and Free Surface Energy Determination

In our study we used the Drop Shape Analyzer Kruss DSA100M optical contact angle measuring instrument (Hamburg, Germany, GmbH) for the evaluation of contact angles. The detailed methodology of experiments, as well as the surface free energy analysis, were presented in our previous paper [28]. We used the Owens–Wendt method [29], which is generally accepted as the best for polymer evaluation. An exact and detailed introduction to the Owens–Wendt methods was presented by Rudawska and co-workers [30]. All the measurements were performed in a special environmental cell at constant temperature conditions (22 °C ± 0.3) and humidity. For each foil sample, at least three successive tests were carried out.

### 2.5. FTIR-ATR Spectrophotometry of Composites

The FTIR–ATR spectra of the composites were recorded in the range of 4000–7000 cm^−1^ using a MATTSON 3000 FT-IR (Madison, Wisconsin, USA) spectrophotometer. This instrument was equipped with a 30SPEC 30 Degree Reflectance adapter fitted with the MIRacle ATR accessory from PIKE Technologies Inc., Madison, Wisconsin, USA.

### 2.6. Thermal Analysis of Composites

Approximately 4 mg of each composite sample was weighed and sealed into aluminium pans. Subsequently, the samples were heated from 25 °C to 400 °C at a rate of 10 °C/min. The empty pan was used as a reference. The tests were performed with the DSC 204F1 Phoenix differential scanning calorimeter (Netzsch company, Selb, Germany). The parameters of the observed thermal transition were calculated with Proteus Analysis software (Netzsch company, Selb, Germany). The analyses were performed in triplicate.

### 2.7. Surface Colour Measurements

The measurement of surface colour was carried out with the use of Konica MINOLTA CM-3500d equipment (Konica Minolta Inc., Tokyo, Japan), with a 30 mm diameter window, using reference D65 illuminant/10° observer. The results were expressed using the CIELab system. The following parameters were determined: L* (L* = 0 black, L* = 100 white), a* - share of the green colour (a* < 0) or red (a* > 0), b*- share of blue (b* < 0) or yellow (b* > 0). The measurements were taken on a standard white background. The experiment was repeated five times.

### 2.8. Thickness Measurement

The thickness of composites was measured with a micrometer, catalog no. 805.1301 (Sylvac SA, Crissier, Switzerland), with a 0.001 mm resolution. The sample thickness was the average of five measurements performed in various places within the gauge length area.

### 2.9. Transparency of Composites

The transparency of composites was measured by exposing the film specimen to light absorption at a wavelength of 600 nm by the use of a UV–vis spectrophotometer (type Helios-Gamma 100-240). Rectangular film samples were placed directly into a spectrophotometer test cell and the empty test cell was used as a reference. The transparency (T) of the films was calculated by the equation

T = A_600_/x (1)

where A_600_ is the absorbance at 600 nm and x is the film thickness (mm). A higher value of T indicates a lower degree of transparency.

### 2.10. UV–Vis Absorption Spectrophotometry

The UV–vis absorption spectra were recorded with a Shimadzu 2101 scanning spectrophotometer in the range of 200–800 nm using 10 mL, 10 mm-thick quartz cells.

### 2.11. Scanning (SEM) and Transmission Electron Microscopy (TEM)

The morphology of as-prepared nanocomposites was studied using a high resolution JEOL JSM – 7500 F Field Emission Scanning Electron (Akishima, Tokyo, Japan) equipped with a Transmission Electron detector (TED).

### 2.12. Water Content, Solubility, and Swelling Degree of Composites

Water content, solubility, and the degree of swelling for films were determined according to the Souza et al. [31] procedure. Briefly, composites were cut into a rectangle specimen (2 × 2 cm^2^) and weighed in analytical balance obtaining initial weight of sample (M1). Then specimens were dried at 70 °C in an oven for 24 h, the initial dry mass (M2) was then analyzed gravimetrically. The samples were then dissolved in 30 mL of distilled water for 24 h at 25 °C. Afterwards the specimens were superficially dried using filter paper and weighed (M3). The residual film samples were dried at 70 °C for 24 h in an oven and the final dry mass was determined (M4). The analysis was performed in tetraplicate replications. Water content, solubility, and degree of swelling of films were calculated according to the equations

Water content (%) = (M1 − M2)/M1 × 100(2)

Solubility (%) = (M2 − M4)/M2 × 100(3)

Swelling degree (%) = (M3 − M2)/M2 × 100(4)

### 2.13. Mechanical Properties of Composites 

Dry composites were conditioned in desiccators at 25 °C and 52% relative humidity (RH) by using saturated solutions of magnesium nitrate-6-hydrate for 48 h prior to analysis. The samples for textural analysis were prepared according to ISO standards [32] and determined using the TA-XT plus texture analyser (Stable Micro Systems, Haslemere, UK). Films were cut into 35 × 6 mm^2^ strips and put into holders. The initial grip separation between holders was 20 mm and the rate of grip separation was 2 mm/min. Tensile strength (TS) was calculated by dividing tensile force (maximum force at rupture of the film) by the cross-section area of the film. The percentage of elongation at the break (EAB) was calculated by dividing the elongation at rupture by the initial gauge length and multiplying by 100. The reported results were the average values of 10 replications.

### 2.14. Cytotoxicity Analysis

#### 2.14.1. Cell Culture Experiments

HepG2 human hepatocellular carcinoma cell line was bought from ATCC (#HB-8065) and were cultured in DMEM low glucose medium (Biowest) supplemented with 10% heat-inactivated fetal bovine serum (FBS, Eurx, Poland), a mixture of antibiotics and antimycotics (penicillin 50 U/mL, streptomycin 50 μg/mL and amphotericin B 250 ng/mL, Corning, USA) and 2 mM glutamine (Corning, Tewksbury, MA, USA). A spontaneously immortalised human keratinocyte cell line HaCat (a gift from Agnieszka Wolnicka-Głubisz, PhD, Jagiellonian University, Cracow, Poland) was maintained in an RPMI 1640 medium (Corning, Tewksbury, MA, USA) supplemented with antibiotics, 10% FBS and 2 mM glutamine as above. Both cell lines were kept at 37 °C with a 5% CO_2_ atmosphere.

#### 2.14.2. Cell Viability Assessment

The HepG2 and HaCat cells were seeded on 96-well plates (2000 cells per well) in 100 μL of culture medium and left to attach for 24 h. The circular pieces of the tested composites, 6 mm in diameter (corresponding to the well size) were cut with a hole puncher, placed in sterile cell culture dishes and sterilised for 2 h under UV light. After sterilisation the composite circles were placed in the wells with the cells, then submerged in the medium. A non-treated control received no composites. After a 48 h incubation period they were removed and cell viability was determined using a CellTiter 96® Aqueous kit (Promega, Germany), which enables spectrophotometric monitoring of MTS tetrazolium reduction by viable cells. Two independent experiments were performed, each in tetraplicate.

### 2.15. Microbiology

A study was carried out on the strains from the ATCC and NCTC collections:

- *Staphylococcus aureus* ATCC 25923
- *Pseudomonas aeruginosa* ATCC 27853
- *Enterococcus faecalis* ATCC 29212
- *Escherichia coli* ATCC 8739
- *Proteus mirabilis* NCTC 11938

Bacterial suspensions with an optical density of 0.5 McFarland were used. The Mueller–Hinton (MH) medium (BioMaxima, Poland) with a volume of 16 cm^3^ were poured into sterile plastic Petri dishes with a diameter of 90 mm. After solidification of the MH medium, 200 μL of bacterial suspensions were spread on the surface with a sterile swab. The circular pieces of tested film (0.5 cm diameter) were placed on the culture surface. Three discs were placed on each Petri dish. Petri dishes were incubated 48 h in 37 °C, aerobic atmosphere. After 48 h incubation, a measurement of the inhibition zone of growth was made. Three independent experiments were performed for all the samples.

### 2.16. Statistical Analysis

The experimental data was subjected to an analysis of variance, at the confidence level of *p* = 0.05, using Statistica v. 8.0 software (Statsoft, Inc., Tulsa, OK, USA). A Fisher test was used for the determination of statistically significant differences.

## 3. Results and Discussion

### 3.1. Zeta Potential and the Particle Size of Composites

All the tested composites possessed a positive zeta potential in the range between ca. 28 mV and 52 mV (Table 1). The zeta potential did not depend on the sample thickness. The lowest zeta potential (about 30 mV) was observed for nanocomposite I. At the same time, the control composite (composite C) and nanocomposite II had higher zeta potential values, reaching over 50 mV.

The analysis of particle size is more complicated. Particle size appeared to depend on composite thickness. The largest particles, about 2000–2500 nm, were observed in the thin composites, while in the thick samples, the particle size was about 600–900 nm.

### 3.2. Contact Angle and Surface Free Energy

Regardless of the concentration, the addition of GO increased the hydrophilic properties of the thin samples (contact angles decrease from 70° to ca 45°, Table 2). The effect was precisely the opposite in the thick composites, where contact angles increased (hydrophobicity rises) from ca. 54° to 80°. Simultaneously, all the composites (independent of GO presence or absence) had almost the same contact angles as diiodomethane (in the range between 30° and 50°).

The surface free energy analysis indicated that the polar surface free energy variations were the most distinguished feature of the tested composites (Table 2). The polar energy strongly depended on the thickness and reached the minimal value in thick samples at the highest GO concentration (nanocomposite I). In thin samples, the effect was the opposite, and polar energy was proportional to GO concentration. The variations of dispersive energy were negligible and did not reveal any particular trend.

### 3.3. FTIR-ATR Spectrophotometry of Composites

**Figure 2:**
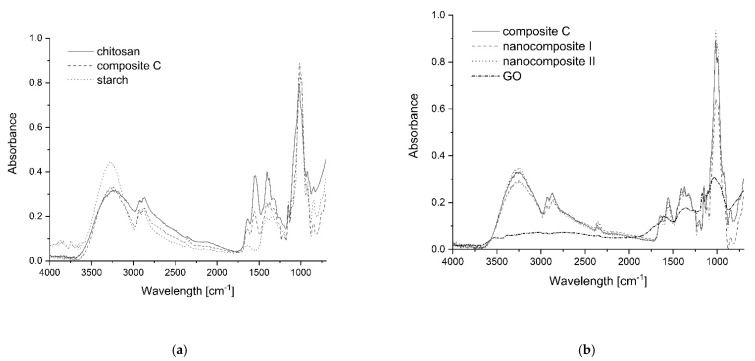
FTIR spectra of (**a**) starch, chitosan and starch/chitosan composites, (**b**) graphene oxide, thin composites: control composite C, nanocomposites I and II.

FTIR spectroscopy was used to examine the interactions between chitosan and starch (Figure 2a). The presence of the broad band at 3251 cm^−1^ in the chitosan spectrum demonstrated the OH stretching and its overlap with the NH stretching in the same region. The band at 1578 cm^−1^ indicated NH (amide II) bending. The peak near 1655 cm^−1^ probably represented the carbonyl group stretching (amide I). In the starch spectrum, the presence of the broad band at 3350 cm^−1^ was associated with OH stretching. The multiple bands between 950–1150 cm^−1^(Figure 2a) corresponded to the asymmetrical vibrations of the C-O-C bridge bonds (1150 cm^−1^), the asymmetric vibrations of the ring (around 1100 cm^−1^) and stretching vibrations of the C-O bonds (the range of 960–1080 cm^−1^). The spectra of both samples (starch and chitosan) revealed multiple bands at 2916–2936, 2855, 1405–1465, and at 1245 cm^−1^ which come from the -CH_2_- group, as well as at 2880–2900 and 3200 cm^−1^ bands, which represented the C-H groups within the polysaccharide molecules. The spectrum of chitosan/starch composite film showed that the addition of starch caused the shift of the chitosan amino peak from 1578–1584 cm^−1^. This result indicated the interactions between the hydroxyl groups of starch and the amino groups of chitosan. The peak of the hydroxyl groups could not be used to evaluate the interactions because of the masking effects of glycerol content [33].

The FTIR spectra of PS/Ch composite (control sample) and GO nanocomposites (Figure 2b) displayed characteristic FTIR peaks corresponding to GO oxygen functionalities, including the C=O stretching vibration peak at 1731 cm^−1^, the C-O (epoxy) stretching vibration peak at 1227 cm^−1^, the C-O (alkoxy) stretching vibration peak at 1065 cm^−1,^ and the vibration and deformation peaks of O-H groups at 3412 cm^−1^ and 1627 cm^−1^ respectively [34]. Generally, the spectra of composite C (control sample) and graphene oxide composite films were very similar, and no significant band shifts could be noted except for a slight difference in the peak intensities, which may have indicated hydrogen bond formation between the components and different water content.

### 3.4. Thermal Analysis of Composites

**Figure 3:**
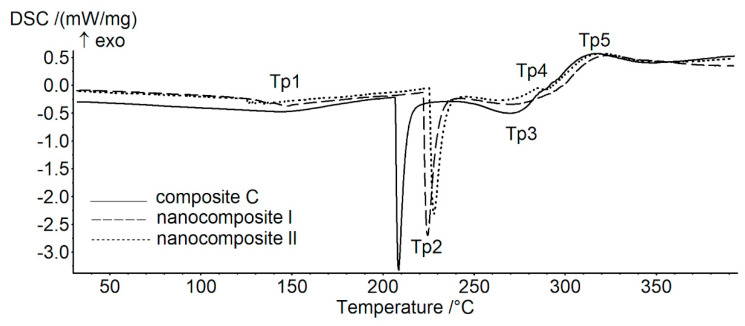
DSC curves of thin control composite C, and nanocomposites I and II.

The DSC curves (Figure 3) demonstrate the presence of five characteristic peaks in the studied temperature range. The first peak (Tp1) is probably related to the occurrence of the glass transition, but it might be disturbed by the relaxation and thermal history of the sample. In other cases, the peaks were most likely related to the melting phenomenon. The temperature values of the observed peaks were determined (Table 3), the largest peak was fully characterised (characteristic temperatures and enthalpy, Table 4). The lowest temperature of the first phase transition (Tp1) was noted for the sample with the highest GO content (nanocomposite I), this was the only sample that differed significantly from the control composite. In the first melting point (Tp2) both samples with GO had significantly higher melting temperatures as compared to the control (Table 4). The temperatures of the other peaks (Tp3 and Tp5) did not differ significantly among the samples. Interestingly, the transition manifested by peak 4 (Tp4) was present only in the sample with the higher GO content (nanocomposite I).

The estimated enthalpy change (ΔH) of melting for nanocomposite I was significantly higher than in the other samples (Table 4). These results indicate that GO alters thermal resistance of the composite films, but the effect is significant only for the highest GO content.

### 3.5. Surface Colour and Transparency

Detailed colour and transparency analysis of the composites confirmed their high transparency and pale yellow hue (Figure 1, Table 5). The incorporation of GO nanoparticles contributed to a statistically significant increase in the parameter of T reaching 1.54 for thin and 1.12 for thick nanocomposites, and consequently a decline in transparency. Surprisingly, the thinner films were less transparent than their thicker counterparts. This may have resulted from the more compact molecular structure of the thin composites. This notion is supported by particle size measurements obtained from DLS (Table 1). The particle size of thin films was double that of thick ones. Water evaporation during the drying of thick composites caused denser compaction of molecules and their flattening due to the limited space. The opposite was the case for the thin composites which had more free space that facilitated a better distribution profile of the polymer particles within the composites.

The values of the L* parameter remained high for both the control and nanocomposite samples, this indicated brightness (Table 5). The incorporation of graphene nanoparticles to the structure of the composite resulted in a decrease of brightness, this was proportional to GO content. Moreover, thin nanocomposites were on average about 13% brighter (higher L * value) than their thicker counterparts. All the nanocomposites had a higher share of red (a* > 0) and yellow (b* > 0) colour than the control composites. The addition of GO nanoparticles increased the a* and b* values significantly (p < 0.05), indicating a tendency towards redness and yellowness. The origin of starch affected the film properties as well. The films composed of corn starch and chitosan were less transparent and darker [35] as compared to the potato starch/chitosan films described here. Fat presence in the corn starch caused opacity and turbidity of gels, whereas potato starch gels were transparent due to marginal fat content. Compared to published examples, our nanocomposite films seem to more transparent than the commonly used synthetic films LDPE (low-density polyethylene) and PVDC (polyvinyl dichloride) [36]. The transparency of composites intended for the production of films and coatings is an important parameter, especially when purchasing behaviour depends on the visibility of a packaged product. On the other hand, such packages should not be used for products requiring UV protection (e.g. products containing fatty acid).

### 3.6. UV–Vis Absorption Spectra

**Figure 4:**
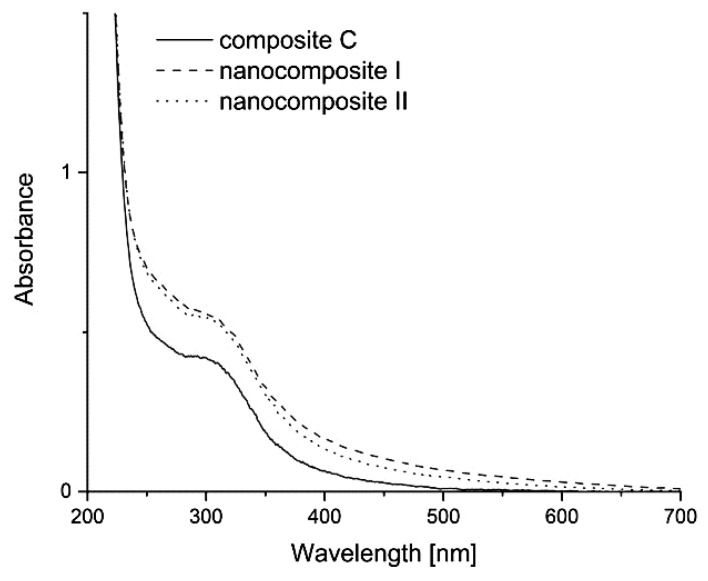
UV–vis spectra of thin composites of control C, nanocomposites I and II.

The GO UV–vis absorption spectrum presented a characteristically sharp absorption peak at about 233 nm and a broad shoulder at 290 nm–305 nm (Figure 4). The peak at 230 nm –235 nm can be attributed to π → π* conjugations of C = C bonds, the peak at 300 nm is n → π* conjugations of C = O bonds [37]. The spectrum of composite C showed a peak at ~310 nm [38]. Because of the fact that the concentration of GO in the composites was relatively low, its absorption peak was hidden under the composite C peak. For this reason, it was difficult to detect the peak for GO in the analysed composites. The π–π interactions between composite C and GO cannot be confirmed from the UV spectra at present, but the nanocomposites exhibited a higher UV absorbance when compared with composite C.

### 3.7. Scanning and Transmission Electron Microscopy

**Figure 5:**
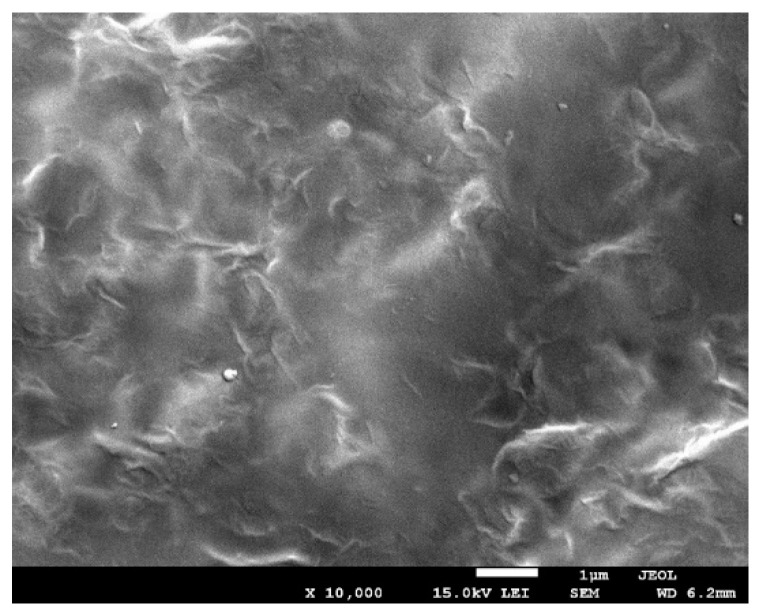
SEM micrograph taken at 10,000 × magnification of thin nanocomposite I.

**Figure 6:**
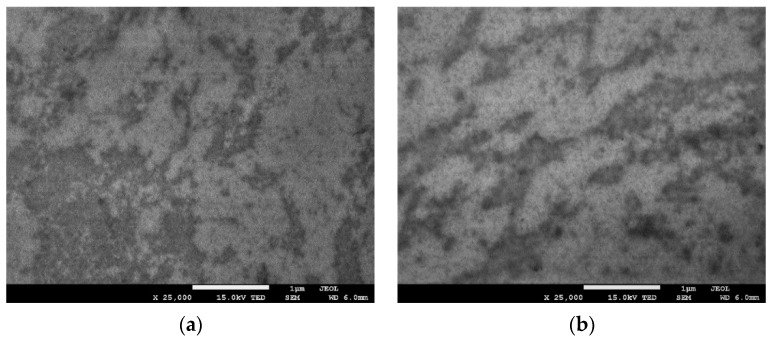
TEM micrograph taken at 25,000 × magnification of (**a**) thin nanocomposite I and (**b**) thin nanocomposite II.

The morphology of chitosan/starch-based nanocomposites containing graphene oxide (GO) is shown in Figure 5 and Figure 6. The TEM and SEM images show well-distinguished GO plate particles in the polysaccharide composite, which means that the graphene oxide has been successfully assembled into polymers [39,40]. The presence of regularly spaced black sheets indicates that GO has been uniformly dispersed throughout the nanocomposite film and that GO does not aggregate in the polysaccharide matrix. It is a very important feature, because GO usually tends to aggregate due to strong inter-planar attractive forces. Uneven distribution of nanoparticles and their aggregation could severely impair the antimicrobial function of the nanocomposites.

### 3.8. Water Content, Solubility, and Degree of Swelling of Composites

The water content in all the composites does not vary much and ranges from 8.81% to 10.92% (Table 6). A descending trend was observed for thick nanocomposites I and II, which resulted from an increase in solid content of GO incorporated in the samples. These values correspond with the thickness measurements shown in Table 7.

The solubility of the thick samples (36.29–37.42%, Table 6) is higher than in their thin counterparts (27.80–30.52 %). The solubility increase is probably caused by the washing out of a portion of the unbound glycerol from the composite structure. The solubility of chitosan in water is limited by its strong intermolecular and intramolecular hydrogen bonds and semi-crystalline structure [41]. On the one hand, the increased dissolving power of the nanocomponent may have a positive effect on its biodegradability, accelerating the decomposition rate in the natural environment. The solubility of the nanocomponent is desirable in cases of using it as a coating or film, particularly when this determines the speed and degree of release of the package contents to another medium. This is important in pharmacy and other biomedical applications. On the other hand, the use of film with high solubility may be limited, especially in the case of solid products with a high water or liquid content, this is due to the penetration of the film components into food or medications.

No statistically significant differences were observed in the degree of swelling of samples with the same thickness (Table 6), this proves that the introduction of graphene oxide does not block some active groups for water absorption. The degree of swelling of the tested films can be lowered by the addition into the matrix of an ingredient that reduces the number of hydrogen bonds to water [29]. The degree of swelling is particularly important for polymer matrix used as a component of medical products (e.g., wound dressing) or food packaging (e.g., lining to absorb product spills). 

### 3.9. Mechanical Properties of Composites

The thickness of the samples varied from 0.095 mm to 0.219 mm (Table 7). The addition of GO increased the composite films’ thickness by approx. 7–12%, which resulted from the enrichment of the solid content in the samples (nanocomposites I and II).

The GO addition improved the EAB by 56–59% and 22–26% for thin and thick nanocomposites respectively (Table 7). The obtained EAB values were double those of Ch/GO reported by Cobos et al. [42]. Besides, the authors demonstrated that Ch/GO composites with added glycerol, had much stronger stretching properties than the composites without it. Being a plasticizer, glycerol modifies thermal and mechanical features of polymers and improves elasticity through maintaining integrity, therefore it protects the matrix from porosity and breakage.

No enhancement in the TS of GO enriched nanocomposites was observed (Table 7). However, it cannot be ruled out that the addition of graphene nanoparticles strengthens the structure of the polysaccharide films. Our previous studies have shown that supplementation with GO improved both the breaking strength and flexibility of the potato starch film [43]. This resulted from high dispersion of GO in the starch matrix and strong interaction between components. Similarly, in the case of carboxymethyl cellulose (CMC) GO composites, a decrease of tensile strength and an increase of elongation at break (by 49%) was observed, this was explained by an increase in elastic stability of polymers after GO incorporation to the CMC foils [43]. The literature suggests that the TS value of thin nanocomposites with GO are comparable with commodity plastic films such as HDPE (22–23 MPa) and LDPE (19–44 MPa) [44].

We have observed that thin composites were 2.5-fold more resistant to breakage (at approx. 21 MPa) than their thicker counterparts. The thin films were comprised of larger particles (Tab. 1). We speculate that much stronger intermolecular forces exist in thin composites, this is what makes them much more resistant to breakage. The DLS results confirm that in the thick films, the interactions among GO, S, and Ch are much weaker: the particle size is smaller after dissolution and the whole system is less dense.

Tensile strength and elongation at break of composites are important parameters for assessing the ability to maintain the integrity of the composites in the presence of environmental stress factors. Therefore, these parameters determine the particular application of such nanomaterials in packaging and other industries [45]. High tensile strength is appreciated, especially in applications where a material should provide structural integrity or reinforce the structure of the packaged products, therefore, deformability is not desirable [35].

### 3.10. Cytotoxicity of Composites

**Figure 7:**
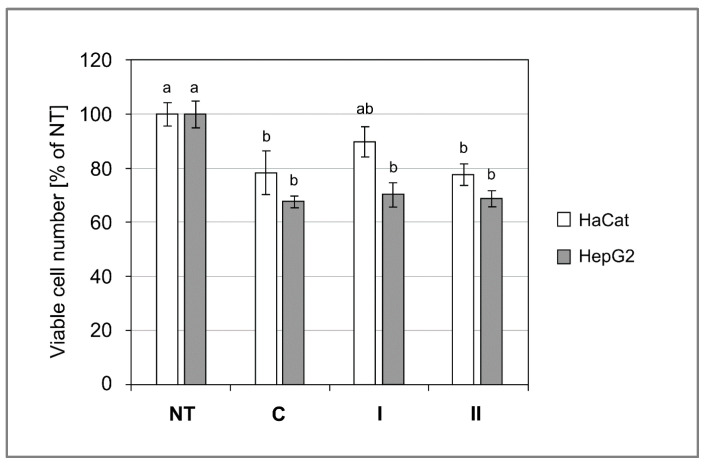
Graphene additive to starch/chitosan composites do not increase cytotoxicity. The bars represent mean ± SEM (*n* = 8) of cell numbers expressed as percentage of non-treated control (NT). C—control composite; I—nanocomposite I.; II —nanocomposite II. The bars with the same letters (a, b) do not differ significantly at the level of confidence 0.05.

The cell viability assays demonstrated that incubation with graphene nanocomposites is well tolerated by human skin keratinocytes. A slowdown of the cell proliferation rate was noticed, but a significantly lower number of viable cells as compared to the non-treated control was observed only for nanocomposite II (Figure 7). Human liver-derived cell line HepG2 was slightly more sensitive than keratinocytes to the composites, showing a roughly 30% lower number of viable cells in the foil-treated groups (Figure 7). However, no signs of cytotoxicity (necrotic floating cells, cell debris, and altered morphology) were observed. In both cell lines there were no significant differences between control composite and graphene nanocomposites. It is likely that a lower number of viable cells in the composite-treated cultures resulted from slowdown of the proliferation rate, which might have been caused by the hampered gas (O_2_, CO_2_) flow and exchange by the film pieces that covered the cultures.

This lack of cytotoxicity is very important, because numerous previous studies reported a considerable level of cytotoxicity in various cell lines [46]. One of the most frequently mentioned mechanisms of GO cytotoxicity is damage of the plasma membrane and intracellular membrane systems [47,48]. Sharp edges of GO flakes and the small size of GO nanoparticles are responsible for such an action [49]. A recent study by Gies et al. [49] analysed the impact of various GO treatments (i.e., base washing, sonication, and cleaning) on cell cytotoxicity. They found that GO nanoparticle size and morphology had a strong impact on cytotoxicity, they further discovered that the majority of their GO preparations decreased viability of cell cultures (A549, U87, and HL-60 cell lines) below 10% at a concentration of 200 μg/mL. Interestingly, HepG2 cells were more resistant, their viability was around 75% for the highest concentration of GO (200 μg/mL), this is similar to our results for this cell line (Figure 7). Nevertheless, our experimental setting was different and the cells were exposed to a much higher concentration of GO (approximately 852.7 μg/mL for nanocomposite I, and 594.7 μg/mL for nanocomposite II), but GO was incorporated within PS/Ch matrices. The GO dispersed within the polysaccharide matrix had little contact with cells, which enabled the cells to tolerate GO content well over four times higher.

### 3.11. Antimicrobial Properties

The bacteriostatic activity of the graphene-containing nanocomposites has been confirmed in *Escherichia coli, Proteus mirabilis*, and *Staphylococcus aureus* cultures, where the growth inhibition zone had 5 mm diameter. The growth inhibition was independent of the graphene concentration. In the case of *Enterococcus faecalis* the growth inhibition was stronger for the samples with lower graphene concentration (0.3 mm) as compared to the control. In the *Pseudomonas aeruginosa* cultures, the GO content did not enhance bactericidal effect, nevertheless, bacterial growth inhibition in the presence of all the composites was clearly visible (Table 8). This inhibitory effect might be attributed to the antimicrobial activity of chitosan. Several mechanisms of bactericidal GO action have been described [11], which include: (i) membrane stress, physical damage, and perforations of bacterial plasmatic membranes exerted by the interactions of amphipathic GO nanoparticles with phospholipids; (ii) GO-induced generation of reactive oxygen species (ROS) that inflict oxidative damage to various biomolecules and are particularly effective against anaerobic and facultatively anaerobic bacterial species; and (iii) photothermal generation of near infra-red radiation that can locally increase temperature. In our study, the first two mechanisms—i.e., the membrane-directed action and oxidative stress—are the most likely responsible for the bacteriostatic effect of the GO-containing nanocomposites. It should be stressed that bacterial cells are much more sensitive to membrane-disruptive agents than eukaryotic cells. Prokaryotes (bacteria) are not equipped with sophisticated membrane repair mechanisms present in eukaryotes and they do not possess complex intracellular membrane systems that are reservoirs of structural phospholipids, nor are they equipped with efficient inter-compartment vesicle transport that secures the transport of lipid cargo to the local membrane injuries [50]. Therefore, it is likely that physical contact with GO-containing nanocomposites leads to material membrane disruption. One may speculate that such a mechanism should be particularly efficient against biofilm-forming pathogenic bacterial species. Alternatively, the GO capacity to generate ROS might create effective means against anaerobic or facultatively anaerobic bacteria that reside in oxygen-deficient niches.

## 4. Conclusions

Here we have described a green synthesis preparation of new bionanocomposites, enriched with GO nanoparticles and based on binary starch/chitosan matrix. We checked how graphene content and film thickness affected their physicochemical and mechanical parameters. Electron microscopy SEM and TEM analysis on the nanocomposites illustrated an even distribution of GO layers within the polysaccharide matrix, which indicated homogenous GO distribution and lack of GO aggregates. The particle size measurements performed with DLS technique confirmed that thin composites had more than double the particle size of their thick counterparts. The higher GO content led to the decrease in zeta potential values and increased EAB values, which indicates improved elasticity. The DSC analysis confirmed the effect of GO on the thermal characteristics of nanocomposites as well as the higher melting temperatures determined in the GO containing samples. Incorporating graphene into S/Ch matrix slightly altered film colours, but the samples were still transparent. All the tested composites were highly permeable regardless of the GO content, which is an advantage in the aspect of biodegradability, although it may limit certain applications. The mechanical studies revealed that thin films were more resistant to rupture. Both thick and thin films showed high elasticity/expandability. The introduction of GO considerably improved nanocomposite elongation at break. The GO-containing nanocomposites exhibited bacteriostatic activity against *Escherichia coli, Proteus mirabilis*, and *Staphylococcus aureus*, but importantly, the lack of toxicity towards human cells was confirmed, which is a great advantage and broadens the range of possible applications in various branches of industry. Collectively, the obtained results indicate the potential application of these nanocomposites as active packaging materials. Further experiments could be designed to test these materials for the storage of various food products.

## Figures and Tables

**Table 1:** Zeta potential and the particle size of composites.

Sample		Particle Size (nm)	Zeta Potential (mV)
composite C	thin	2545	50.5
nanocomposite I		1935	36.5
nanocomposite II		2140	40.0
composite C	thick	620	56.2
nanocomposite I		880	28.3
nanocomposite II		590	51.8

**Table 2:** Contact angle and surface free energy of the composites.

Sample		Contact Angle		Surface Free Energy		
		Water	Diiodomethane	Polar (mJ/m^2^)	Dispersive (mJ/m^2^)	Total Free Energy (mJ/m^2^)
composite C	thin	70.0°	45.9°	9.140	34.14	43.28
nanocomposite I		45.7°	53.5°	29.96	23.76	53.72
nanocomposite II		47.7°	33.8°	21.31	35.86	57.17
composite C	thick	53.8°	43.6°	19.65	31.57	51.23
nanocomposite I		80.2°	40.0°	3.15	41.08	44.23
nanocomposite II		64.1°	39.1°	11.25	36.91	48.16

**Table 3:** Temperature values of characteristic peaks.

Sample	Tp1	Tp2	Tp3	Tp4	Tp5
	°C	°C	°C	°C	°C
composite C	142.2 ± 2.3 ^b^	215.5 ± 6.4 ^a^	265.2 ± 5.2	nd	318.6 ± 1.7
nanocomposite I	123.2 ± 3.2 ^a^	226.4 ± 1.5 ^b^	267.8 ± 6.3	284.9 ± 0.8	320.8 ± 1.1
nanocomposite II	141.2 ± 9.1 ^b^	223.9 ± 0.7 ^b^	273.8 ± 7.1	nd	322.6 ± 2.1
One-way ANOVA - p	0.011	0.029	0.297	-	0.068

Mean value (*n* = 3) ± SD. Parameters in columns denoted with the same letters (a. b. c. etc.) do not differ statistically at the level of confidence 0.05.

**Table 4:** Parameters of melting peak (Tp2).

Sample	Ton	Tp	Tend	-ΔH
	°C	°C	°C	J·g^−1^
composite C	213.0 ± 5.6 ^a^	215.5 ± 6.4 ^a^	222.6 ± 8.3	88.1 ± 1.1 ^b^
nanocomposite I	224.0 ± 1.6 ^b^	226.4 ± 1.5 ^b^	232.6 ± 1.5	81.0 ± 3.2 ^a^
nanocomposite II	221.7 ± 0.5 ^b^	223.9 ± 0.7 ^b^	229.7 ± 1.2	87.5 ± 2.4 ^b^
One-way ANOVA - p	0.015	0.029	0.109	0.020

Mean value of three replication ± standard deviation. Parameters in columns denoted with the same letters (a. b. c. etc.) do not differ statistically at the level of confidence 0.05. Tp—peak temperature, Ton—onset temperature, Tend—end set temperature, ΔH—melting enthalpy

**Table 5:** Colour parameters and transparency of composites.

Sample		L* (D65)	a* (D65)	b* (D65)	T (A·mm^−1^)
composite C	thin	97.68 ± 0.03 ^a^	−0.91 ± 0.01 ^e^	6.63 ± 0.08 ^f^	1.21 ± 0.10 ^b^
nanocomposite I		83.31 ± 0.87 ^d^	1.13 ± 0.13 ^c^	12.66 ± 0.34 ^d^	1.54 ± 0.07 ^a^
nanocomposite II		88.49 ± 0.25 ^c^	0.27 ± 0.01 ^d^	11.76 ± 0.22 ^e^	1.49 ± 0.05 ^a^
composite C	thick	95.35 ± 0.16 ^b^	−2.03 ± 0.02 ^f^	16.47 ± 0.37 ^c^	0.57 ± 0.01 ^c^
nanocomposite I		71.98 ± 1.24 ^f^	2.41 ± 0.26 ^a^	21.22 ±1.01 ^b^	1.12 ± 0.04 ^b^
nanocomposite II		77.46 ± 0.45 ^e^	1.51 ± 0.08 ^b^	22.10 ± 0.53 ^a^	0.95 ± 0.06 ^c^

Parameters in columns denoted with the same letters (a. b. c. etc.) do not differ statistically at the level of confidence 0.05.

**Table 6:** Water content, solubility, and degree of swelling of composites.

Sample		Water Content (%)	Solubility (%)	Swelling Degree (%)
composite C	thin	10.92 ± 1.30 ^a^	27.80 ± 1.08 ^c^	95.18 ± 17.88 ^c^
nanocomposite I		8.81 ± 0.42 ^c^	30.52 ± 2.05 ^b^	107.57 ± 7.79 ^bc^
nanocomposite II		9.79 ± 0.75 ^bc^	29.65 ± 0.32 ^bc^	102.49 ± 7.08 ^c^
composite C	thick	10.16 ± 0.22 ^ab^	36.82 ± 0.38 ^a^	127.66 ± 8.31 ^a^
nanocomposite I		9.90 ± 0.59 ^ab^	37.42 ± 3.07 ^a^	121.94 ± 11.17 ^ab^
nanocomposite II		9.43 ± 0.54 ^bc^	36.29 ± 1.01 ^a^	125.15 ± 13.85 ^a^

Parameters in columns denoted with the same letters (a. b. c. etc.) do not differ statistically at the level of confidence 0.05.

**Table 7:** Mechanical properties of composites.

Sample		Thickness (mm)	TS (MPa)	EAB (%)
composite C	thin	0.095± 0.008 ^d^	21.22 ± 4.34 ^a^	37.75 ± 6.12 ^c^
nanocomposite I		0.102 ± 0.005 ^c^	20.98 ± 1.97 ^a^	60.14 ± 5.27 ^ab^
nanocomposite II		0.107 ± 0.008 ^c^	20.49 ± 3.22 ^a^	59.23 ± 8.01 ^ab^
composite C	thick	0.205 ± 0.012 ^b^	8.43 ± 1.79 ^b^	52.50 ± 6.37 ^b^
nanocomposite I		0.216 ± 0.024 ^a^	8.15 ± 1.32 ^b^	66.52 ± 9.98 ^a^
nanocomposite II		0.219 ± 0.009 ^a^	8.10 ± 1.85 ^b^	64.29 ± 8.04 ^a^

TS—Tensile strength, E—Elongation at break. Parameters in columns denoted with the same letters (a. b. c. etc.) do not differ statistically at the level of confidence 0.05.

**Table 8:** Diameter of growth inhibition zone on thin composites: average and standard deviation.

Species of Bacteria	Samples	Average ± Standard Deviation (mm)	Range (mm)
*Escherichia coli (EC)*	composite C	0.0 ± 0.0	0.0–0.0
	nanocomposite I	5.0 ± 0.0	5.0–5.0
	nanocomposite II	5.0 ± 0.0	5.0–5.0
*Proteus mirabilis (PM)*	composite C	0.0 ± 0.0	0.0–0.0
	nanocomposite I	5.0 ± 0.0	5.0–5.0
	nanocomposite II	5.0 ± 0.0	5.0–5.0
*Staphylococcus aureus (SA)*	composite C	0.0 ± 0.0	0.0–0.0
	nanocomposite I	5.0 ± 0.0	5.0–5.0
	nanocomposite II	5.0 ± 0.0	5.0–5.0
*Enterococcus faecalis (EF)*	composite C	9.7 ± 0.6	9.1–10.3
	nanocomposite I	9.0 ± 1.0	8.0–10.0
	nanocomposite II	10.0 ± 1.0	9.0–11.0
*Pseudomonas aeruginosa (PA)*	composite C	11.0 ± 1.0	10.0–12.0
	nanocomposite I	10.0 ± 0.0	10.0–10.0
	nanocomposite II	5.0 ± 0.0	5.0–5.0

## Data Availability

Raw data are deposited in the University of Agriculture Repository.
